# Virulence factors of *Candida species* isolated from patients with urinary tract infection and obstructive uropathy

**DOI:** 10.12669/pjms.321.8559

**Published:** 2016

**Authors:** Faris Q.B. Alenzi

**Affiliations:** 1 Faris Q.B. Alenzi, Dept of Medical Laboratory Sciences, College of Applied Medical Sciences, Prince Sattam bin Abdulaziz University, Al-Kharj, Saudi Arabia

**Keywords:** *Candida*, UTIs, Uropathy, Virulence

## Abstract

**Objective:**

Fungal urinary tract infections due to *Candida* have increased significantly in recent years. Our research objective was to study *Candida species* in urine samples of patients with urinary tract infections (UTIs) associated with obstructive uropathy and to investigate the virulence factors of the isolated *Candida*.

**Methods:**

Patients were divided into two groups: Group I (cases): 50 patients with UTIs and obstructive uropathy. Group II (control): 50 patients with UTIs but with no functional or anatomical obstruction of their urinary tract. Clinical histories and physical examinations, together with laboratory investigations of urine samples were carried out in all patients in this study. Mid stream urine samples were examined microscopically and by fungal cell culture. The isolated *Candida species were* identified by analytical profile index (API). *Candida* Virulence factors were determined for the isolated *Candida*. The susceptibility to fluconazole was evaluated.

**Results:**

This study revealed an overall isolation rate of 27% of *Candida* species among all patient groups. The rate was 36% in cases, and 18% in controls, a difference found to be statistically significant (P<0.05). By API, *C.albicans* was detected in 44% of *Candida* species in cases, and in 33% in controls. While C.glabrata was detected in 28% of *Candida* species in cases, and in 22% in controls. C.tropicalis was detected in 17% of *Candida* species in cases, and in 22% in controls. Both C.krusei and C.kyfr were detected in 5.5% of *Candida* species in cases, and in 11% in controls. In terms of virulence factors the study showed that 11 out of 27 (40.5%) of *Candida* isolates were biofilm positive by tube adherence. Phospholipase activity was demonstrated in 12 out of 27 (44.5%) of *Candida* isolates. Secretory aspartic proteinase activity was demonstrated in 13 out of 27 (48%) of the *Candida* isolates.

**Conclusion:**

*Candida* is an important cause of UTIs and obstructive uropathy is a major predisposing factor.

## INTRODUCTION

Community acquired urinary tract infections (UTIs) are a frequent problem worldwide and are caused by microbial invasion to different tissues of the urinary tract.^1^ Fungi are a portion of the microbial population that may contribute as fungal uropathogens in UTIs. Over the last year, the incidence of fungal UTIs due to *Candida* has increased significantly.^1^ The increased incidence of fungal infections in patients suffering from urinary tract infections associated with obstructive uropathy as well as the emergence of azole antifungal drug resistance justifies an immediate need to investigate mechanisms of *Candida* species pathogenicity and their relationship to drug resistance.^2^

Secreted aspartyl proteinases (SAPs) are extracellular proteolytic enzymes that play a central role in *Candida* pathogencity. SAPs carry out a number of specialized functions during the infection process, which facilitates their adhesion and tissue invasion.*3* Phospholipases are another group of enzymes that contribute to the pathogencity of *C. albicans* by damaging host-cell membranes, and thus facilitating fungal invasion of host tissues.*4*

There is a direct relationship between the adherence of *Candida* species and their ability to colonize the biomaterial. The species which are more adherent are those which are more virulent.*2* Therefore, rapid and reliable identification of *Candida* species and their virulence factors represents an important factor in routine clinical microbiology practice.

The aim of this work was to study different *Candida* species in urine samples from patients with urinary tract infections associated with obstructive uropathy, and to investigate the virulence factors of the isolated *Candida* species including; biofilm formation, the secreted aspartic proteinase and the extracellular phospholipase activity.

## METHODS

This study was carried out on urine samples obtained from 100 patients attending primary health care centers, Arar, KSA. Patients were classified into two groups: Group I: included 50 patients with UTIs and obstructive uropathy. They comprised 18 males and 32 females. Their ages ranged from 15 to 58 years. Group II (controls): included 50 patients with UTIs and who had no functional or anatomical abnormality of urinary tract. They comprised 20 males and 30 females. Their age ranged from 14 to 53 years. This study was conducted at the Northern Borders region of Saudi Arabia (Arar) and other private centers in the region, Saudi Arabia between Jan-2013 to Jan-2015. Written informed consent and Research Ethics Committee approval were obtained in all cases.

### Patients Received

***I. An assessment of their clinical history:*** Previous urologic problems, operation, terminal haematuria, dysuria and taking of antibiotics, or any other medications.

***II. Clinical and radiological examination; III. Laboratory investigations of urine samples:*** Including Mid-stream urine samples or catheterized urine samples were obtained under aseptic conditions in sterile screw-capped wide mouth containers. Samples were subjected to microscopic examination and culture. Identification of *Candida species* by Gram-stain, Germ Tube test, subculture on chromID™ *Candida* Agar (CAN2): (bioMérieux) and biochemically by API *Candida* (bioMérieux).

### Detection of virulence factors of Candida

### Biofilm Formation

Biofilm formation was carried out by tube adherence as by company instructions.

### Phospholipase Activity

Assessment of phospholipase activity was carried out using Sabouraud egg yolk agar plate, in which the phospholipase activity was calculated as “the ratio of the diameter of the colony plus the precipitation zone around it to the diameter of the colony alone”.

Aspartic proteinase detection was carried out using bovine serum albumin (BSA) agar, Disc Diffusion Test was carried out using sterile swabs and plates of Mueller Hinton agar supplemented with 2% glucose.

### Data Analysis

Data were analyzed using the SPSS software (Statistical Package for the Social Science) version 16.

## RESULTS

The current study showed that 27% of the patients (27 out of 100) were *Candida* positive (Table-I). Also, the incidence of *Candida* infection in cases (group I) (obstructive uropathy) was found to be higher than that in controls (group II), i.e. 36% versus 18% (a difference which was statistically significant) (Table-I).

**Table-I:** Candida isolates among studied groups.

*Groups*	*Candida isolates*		*p- value*

	No.	%	
Group-I, cases (n=50)	18	36	<0.05
Group-II , controls (n=50)	9	18	

Total (n =100)	27	27	

*Candida* species were identified by API *Candida*. In group I (cases), *C.albicans* was detected in 44% (8 out of 18), followed by *C.glabrata* in 28% (5 out of 18), and *C.tropicalis* in 17% of cases (3 out of 18). In group II controls, *C.albicans* was detected in 33% (3 out of 9), followed by *C. glabrata* and *C.tropicalis* in 22% in patients (2 out of 9). In both groups, *C.albicans* was detected in 41% of patients (11 out of 27), followed by *C. glabrata* in 26% (7 out of 27), and *C.tropicalis* in 19% in patients (5 out of 27). This study, 41% (11 out of 27) of *Candida* isolates were biofilm positive by tube adherence (Table-II).

**Table-II:** Candida albicans & Candida non albicans among different groups by API.

Patient group	Candida isolates										Total

	C.albicans		C.glabrata		C.tropicalis		C.krusei		C.kyfr		

	No.	%	No.	%	No.	%	No.	%	No.	%	
Cases	8	44.4	5	27.7	3	16.6	1	5.5	1	5.5	18
Controls	3	33.3	2	22.2	2	22.2	1	11.1	1	11.1	9

Total	11	40.7	7	25.9	5	18.5	2	7.4	2	7.4	27

Secretory aspartic proteinase activity was demonstrated in 13 out of 27 (48%) of *Candida* isolates. There were statistically significant differences in biofilm formation phospholipase activity and in proteinase activity between *C.albicans* and *C.non.albicans*. This study, *C.albicans* isolates recovered from urine demonstrated a lower percentage of biofilm positivity than other *Candida* species isolates (20% vs. 53%) (Table-III).

**Table-III:** Comparison between C.albicans & C. non albicans groups as regards to virulence factors.

	C.albicans (n=10)		C. non albicans (n=17)		p

	No	%	No	%	
Biofilm formation	2	20	9	53	<0.05
Phospholipase activity	5	50	7	41.	>0.05
Proteinase activity	7	70	6	35.	<0.05

This study showed that 55% of *C.albicans*, 14% of *C.glaberata*, 60% of *C.tropicalis*, 50% of *C.krusei*, and, 50% of *C.kyfr* were susceptible to fluconazole. In contrast, 27% of *C.albicans*, 71% of *C.glabrata*, 40% of *C.tropicalis*, 50% of *C.krusei*, and 50% *C.kyfr* were resistant to fluconazole (Table-IV).

**Table-IV:** Susceptibility of isolates of *Candida* species as determined by the disc diffusion test to fluconazole.

*Candida* species	Susceptible			SDD*			Resistant		

	Zone diameter	No.	%	Zone diameter	No.	%	Zone diameter	No.	%
C.albicans (n=11)	22-32	6	54	18	2	10	10-12	3	27
C.glabrata (n=7)	28	1	14.3	16	1	14.3	1-12	5	71.4
C.tropicalis (n=5)	20-30	3	60	--	0	0	5-9	2	40
C.krusei (n=2)	35	1	50	--	0	0	5-12	1	50
C. kyfr (n=2)	29	1	50	--	0	0	12	1	50

Susceptible >19 mm,

* Susceptible Dose Dependent (SDD), 15-18 mm, Resistant ≤ 14 mm.

## DISCUSSION

All known *Candida* species are capable of causing UTIs. *C.albicans* was the most common isolated species. However, in many centers worldwide, non-*Candida* albicans species now predominate. The incidence of fungal infections is increasing because the number of immunocompromised patients is increasing; also there is widespread use of broad-spectrum antibiotics and invasive devices or procedures.^5^ These results are in agreement with those of Din et al^6^ isolated *Candida* species from patients suffering from UTIs in 24% and 25%, respectively. In this study, API system has been used here for the characterization of Candida spp, which is only useful upto some extent. However, for research purpose, other alternative molecular methods can be consider as well.

Borst and Fluit^7^ in the Netherlands reported a prevalence rate of 24%, and Kobayashi et al^8^ in Brazil found a prevalence rate of 22%. A higher percentage in obstructive uropathy was also recorded and described by Frkiaer^9^ who found in a retrospective study of patients with genitourinary fungal infection, that obstructive uropathy was associated with 88% of cases.

These results are in agreement with those of Miller^10^ who detected *C.albicans* in 41% of cases, *C. glabrata* in 20%, and *C.tropicalis* in 17% by API system of *Candida* infection isolated from urine samples. Also higher results were also obtained by Alhussaini et al^11^ who detected *C.albicans* in 54%, *C.glabrata* in 16%, and *C.tropicalis* in 6% of patients. Another study, conducted by Achkar and Fries 12 in 2010, found that in data collected from 10 medical centers in US, *C.albicans* was the commonest isolated from 52% of all *Candida species*, *C.glabrata* in 16%, and *C.tropicalis* in 8% of cases.

These findings are in agreement with those of Tortorano et al^13^ who have reported that the biofilm formation was observed in 39% of *Candida* isolates. Phospholipase activity in our study was demonstrated in 12 out of 27 (44%) of *Candida* isolates.

These results are in agreement with those Khun et al^14^ who reported that *C.albicans* isolates recovered from urine demonstrated lower percentage of biofilm positivity than other *Candida* species isolates. Our results also agree with that of Tsang et al^15^ who detected phospholipase activity in 84% of the *C. albican* isolates.

In conclusion, we herein report that *Candida* infection is an important cause of UTIs and obstructive uropathy is a major predisposing factor. The diagnostic and/or prognostic significance of these infections requires further study with a large cohort of patients.
